# NLRC5-CIITA Fusion Protein as an Effective Inducer of MHC-I Expression and Antitumor Immunity

**DOI:** 10.3390/ijms24087206

**Published:** 2023-04-13

**Authors:** Madanraj Appiya Santharam, Akhil Shukla, Dominique Levesque, Thomas A. Kufer, François-Michel Boisvert, Sheela Ramanathan, Subburaj Ilangumaran

**Affiliations:** 1 Department of Immunology and Cell Biology, Faculty of Medicine and Health Sciences, Université de Sherbrooke, Sherbrooke, QC J1H 5N4, Canada; 2 Department of Immunology, Institute of Nutritional Medicine, University of Hohenheim, 70593 Stuttgart, Germany; 3 CRCHUS, Centre Hospitalier de l’Université de Sherbrooke, Sherbrooke, QC J1H 5N4, Canada

**Keywords:** NLRC5, MHC-I, tumor immunogenicity, NLRC5-SA, B16-F10, EL4, MHC-I associated peptides

## Abstract

Aggressive tumors evade cytotoxic T lymphocytes by suppressing MHC class-I (MHC-I) expression that also compromises tumor responsiveness to immunotherapy. MHC-I defects strongly correlate to defective expression of NLRC5, the transcriptional activator of MHC-I and antigen processing genes. In poorly immunogenic B16 melanoma cells, restoring NLRC5 expression induces MHC-I and elicits antitumor immunity, raising the possibility of using NLRC5 for tumor immunotherapy. As the clinical application of NLRC5 is constrained by its large size, we examined whether a smaller NLRC5-CIITA fusion protein, dubbed NLRC5-superactivator (NLRC5-SA) as it retains the ability to induce MHC-I, could be used for tumor growth control. We show that stable NLRC5-SA expression in mouse and human cancer cells upregulates MHC-I expression. B16 melanoma and EL4 lymphoma tumors expressing NLRC5-SA are controlled as efficiently as those expressing full-length NLRC5 (NLRC5-FL). Comparison of MHC-I-associated peptides (MAPs) eluted from EL4 cells expressing NLRC5-FL or NLRC5-SA and analyzed by mass spectrometry revealed that both NLRC5 constructs expanded the MAP repertoire, which showed considerable overlap but also included a substantial proportion of distinct peptides. Thus, we propose that NLRC5-SA, with its ability to increase tumor immunogenicity and promote tumor growth control, could overcome the limitations of NLRC5-FL for translational immunotherapy applications.

## 1. Introduction

Cancer cells are under constant immune surveillance by CD8^+^ cytotoxic T lymphocytes (CTLs), which kill tumor cells expressing neoantigens [1]. CD8^+^ T cells recognize antigenic peptides presented by class-I major histocompatibility (MHC-I) molecules, which are composed of an α chain and β2 microglobulin (β2M) [2,3]. These antigenic peptides are derived from aged and misfolded cellular proteins and polypeptides following cleavage by proteasomes. Recognition of tumor antigenic peptides is crucial for the activation of naïve anti-tumor CD8^+^ T cells and subsequent killing of tumor cells by CTLs [4]. Genomic, proteomic and proteogenomic approaches are used to identify tumor antigenic peptides [5,6,7,8,9]. These tumor antigenic peptides can be exploited for personalized vaccines, expansion of antitumor CD8^+^ T cells for adaptive cell therapy and for the development of chimeric antigen-receptor-bearing T cells [10,11].

Aggressive tumors use diverse strategies to evade the immune system [4,12]. A key immune evasion mechanism is the loss of MHC-I, which allows the tumors to escape CTL-mediated killing as well as impair further CD8^+^ T cell activation [13,14,15]. Loss of MHC-I can arise from irreversible or reversible genetic lesions. The former can result from the loss of MHC-I gene alleles, loci or haplotypes, β2M or any key component of the MHC-I antigen processing machinery (APM) required to generate antigenic peptides [16,17,18,19,20]. Reversible MHC-I defects arise from epigenetic modifications at MHC-I and APM genes [21,22,23,24,25,26]. Several reports have highlighted the need to restore MHC-I expression in cancer cells in order to render them susceptible to CTLs [27,28].

NLRC5 is a crucial transcriptional activator of MHC-I and APM genes [29,30]. TCGA data from diverse cancers revealed that loss of NLRC5 expression is the most common defect among MHC-I-related genes and correlates with reduced CTL infiltration and poor patient survival [31]. In addition, NLRC5 expression was shown to be downmodulated by the lncRNA SCAMP1 (Secretory carrier-associated membrane protein 1) in gliomas [32]. Recently, Zhan et al. reported that immune evasion of endometrial cancers by low MHC-I expression is associated with inhibition of NLRC5 by the autophagy protein LC3 [33]. Even though a pro-tumor role of NLRC5 is proposed for certain cancers [34,35], this needs to be tested in immune-competent settings. Nonetheless, the strong evidence for the MHC-I transactivating function of NLRC5 spurred research on its potential utility for immunotherapy against MHC-I low tumors. We have shown that NLRC5 renders B16F10 mouse melanoma cells highly immunogenic via upregulating MHC-I and APP genes that facilitates the processing and presentation of endogenous tumor antigenic peptides [36]. Other studies have also implicated NLRC5 in promoting antitumor immunity [37,38]. Recent studies show that loss of NLRC5 expression is associated with unresponsiveness to immune checkpoint blockade therapy [39]. These reports raise the possibility of exploiting NLRC5 to increase cancer immunogenicity, strengthen other cancer immunotherapy approaches and possibly identify tumor antigenic peptides.

Even though epigenetic drugs that inhibit DNA and histone methylation can be used to restore MHC-I expression via derepressing *NLRC5* and *MHC-I* genes, their possible off-target effects mandate exploring alternative approaches [22,25,40,41]. One such approach could be introducing the *NLRC5* gene via viral vectors or other gene delivery approaches. However, *NLRC5* encodes a very large protein of 205 kDa [42], which constrains its delivery to tumors. NLRC5 has an N-terminal atypical CARD (aCARD) domain, central NACHT domain and C-terminal leucine-rich repeats (LRRs). NLRC5 is closely related to NLRA, also known as MHC class-II transactivator (CIITA) [30]. We have previously shown that a fusion protein containing the CARD domain of NLRC5 and the central and C-terminal portion of CIITA retained the ability to induce MHC-I [43]. This fusion protein (~100 kDa) was more efficient than full-length NLRC5 (NLRC5-FL) in activating the MHC-I gene promoter in HEK293T cells, and hence dubbed NLRC5 super activator (NLRC5-SA). The smaller size of NLRC5-SA renders it an attractive candidate for exploring its potential to boost cancer immunogenicity via restoring the expression of MHC-I.

In this study, we compared NLRC5-SA and NLRC5-FL for their ability to induce MHC-I expression in mouse and human cancer cell lines, control B16-F10 melanoma and EL4 thymoma growth in syngeneic C157BL/6 mice and modulate in MHC-I-associated peptides (MAPs). Our findings reveal that NLRC5-SA is as efficient as NLRC5-FL in these assays, supporting its potential translational utility.

## 2. Results

### 2.1. Induction of MHC-I Expression by NLRC5-SA in Mouse and Human Cancer Cell Lines

**Figure 1:**
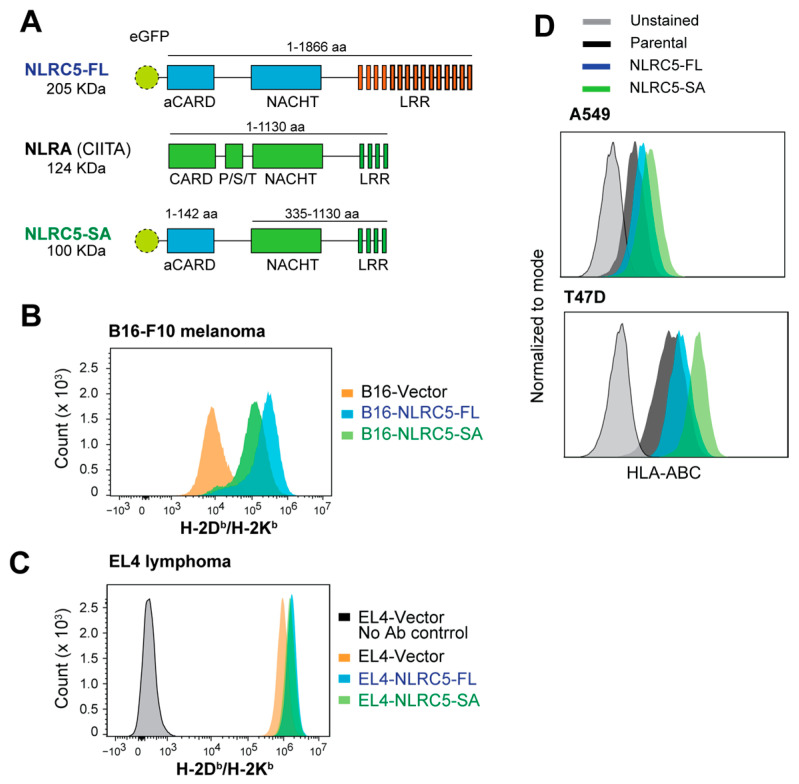
MHC-I induction by NLRC5-SA in mouse and human cancer cell lines. (**A**) The NLRC5-SA construct. NLRC5 superactivator (NLRC5-SA) was generated by fusing the N-terminus atypical CARD (aCARD) domain of full-length human NLRC5 (NLRC5-FL) to the central NACHT and C-terminus LRR regions of NLRA (CIITA), thereby retaining its tripartite structure. (**B**) Cell surface MHC-I expression in mouse B16.F10 melanoma cells stably expressing control vector, NLRC5-FL or NLRC5-SA expression vectors evaluated by flow cytometry using a bi-specific H-2Kb/Db antibody. (**C**) MHC-I expression in mouse EL4 lymphoma cells expressing control vector, NLRC5-FL or NLRC5-SA expression constructs. (**D**) Cell surface expression of HLA-ABC in A549 lung cancer and T47D breast cancer cell lines stably expressing NLRC5-FL or NLRC5-SA labeled with a pan-HLA antibody.

NLRC5-SA harbors the aCARD domain of NLRC5 and the NACHT-LRR domains of NLRA (CIITA) [43] (Figure 1A). Stable expression of NLRC5-SA in B16-F10 (B16) melanoma cells, which express negligible levels of NLRC5 and MHC-I at steady state [36], markedly upregulated the expression of MHC-I molecules H-2Kb and H-2Db, albeit to a discernibly lower level than full-length NLRC5 (NLRC5-FL) (Figure 1B). In contrast to B16 cells, EL4 lymphoma cells display high basal expression of MHC-I and are extensively used to study MHC-I antigen processing and presentation [44,45,46,47]. Stable expression of NLRC5-SA caused a small but significant increase in MHC-I expression compared to EL4-vector cells to the same extent as NLRC5-FL (Figure 1C). Next, we examined the effect of NLRC5-SA in human cancer cell lines selected from the NCI-60 cell line panel based on low basal expression of NLRC5 and HLA genes [29,36]. The NCI-60 panel cells show varying levels of NLRC5 and its target genes HLA-B, TAP1 and PSMB8 (Supplementary Figure S1). Even though NLRC5 expression correlated positively with HLA and APM genes in the NCI-60 cell line panel as a whole (Supplementary Figure S2A), individual cell lines showed considerable variation between NLRC5 and HLA-B (Supplementary Figure S1). Among these cell lines, A549 (lung carcinoma), MCF7 (mammary adenocarcinoma) and T47D (mammary ductal carcinoma) were selected for low NLRC5 expression and varying levels of HLA expression (Supplementary Figure S2B). A549 and T47D were responsive to IFNγ and upregulated NLRC5, whereas MCF7 cells responded poorly and failed to upregulate NLRC5, suggesting possible deletion or copy number loss of the NLRC5 gene. A549 and T47D cell lines were transfected with NLRC5-FL and NLRC5-SA, and stable lines were evaluated for HLA expression (Figure 1D). Efforts to stably transfect MCF7 with NLRC5 constructs were not successful. T47D cells displayed discernibly higher HLA expression than A549 cells. In both cell lines, NLRC5-SA induced HLA protein expression more efficiently than NLRC5-FL did. These results indicate that NLRC5-SA can be exploited to restore MHC-I expression in MHC-I low cancers.

### 2.2. Attenuation of B16 Melanoma and EL4 Lymphoma Tumor Growth by NLRC5-SA

**Figure 2:**
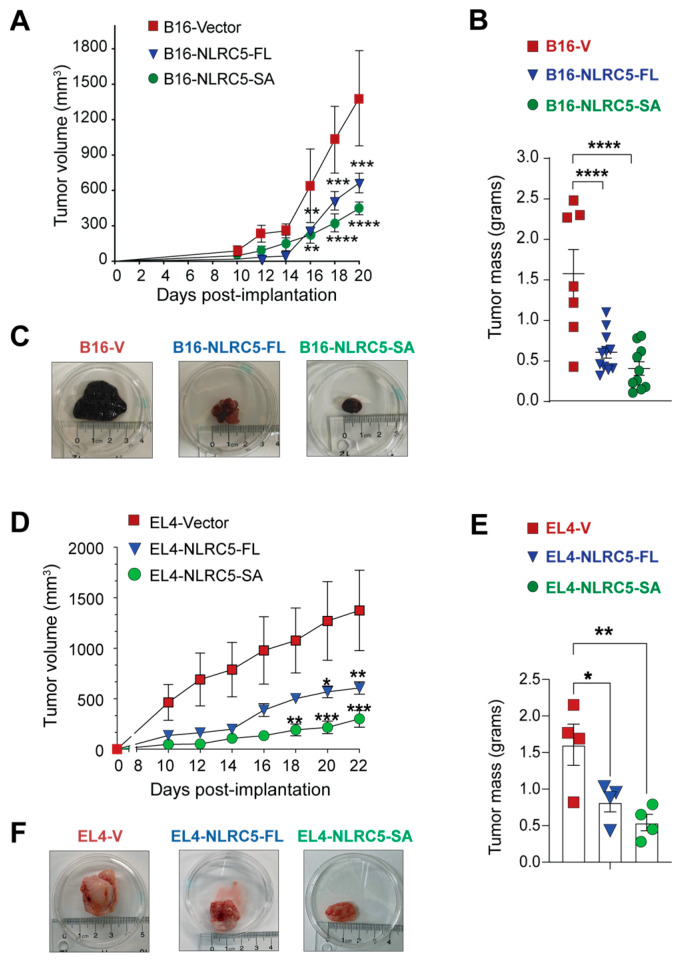
Control of B16 melanoma and EL4 lymphoma tumor growth by NLRC5-SA. Syngeneic C57BL/6 mice were inoculated subcutaneously with 1 × 10^5^ B16-vector, B16-NLRC5-FL or B16-NLRC5-SA (**A**–**C**; *n* = 7–11 per group) or 5 × 10^5^ EL4-vector, EL4-NLRC5-FL or EL4-NLRC5-SA cells (**D**–**F**; 4 mice per group). Tumor growth curve, monitored every 2–3 days (**A**,**D**). Tumor mass at the endpoint (**B**,**E**). Representative tumors formed by each tumor line at the endpoint (**C**,**F**). Statistical comparisons (in **A**,**B**,**D**,**E**): mean ± SE; two-way ANOVA with Tukey’s multiple comparison test. * *p* < 0.05, ** *p* < 0.01, *** *p* < 0.001, **** *p* < 0.0001.

We have previously shown that NLRC5-FL expression in B16.F10 melanoma cells reduces tumor growth by promoting CD8^+^ T cell activation and CTL-mediated tumor killing [36]. As NLRC5-SA upregulates MHC-I as strongly as NLRC5-FL, we evaluated the growth of B16 melanoma and EL4 lymphoma cells expressing NLRC5-SA, NLRC5-FL or NLRC5-control vector as tumors in syngeneic C57BL/6 mice. Even though MHC-I expression induced by NLRC5-SA in B16 cells was discernibly lower than NLRC5-FL-mediated MHC-I induction (Figure 1B), NLRC5-SA expression significantly reduced B16 tumor growth as strongly as NLRC5-FL did (Figure 2A–C). Similarly, NLRC5-FL and NLRC5-SA caused only a small increase in MHC-I expression in EL4 lymphoma cells (Figure 1C), yet EL4-NLRC5-FL and EL4-NLRC5-SA cells formed significantly smaller tumors than EL4-vector control cells (Figure 2D–F).

### 2.3. Acid Elution of MHC-I-Bound Peptides EL4-NLRC5-SA Cells

**Figure 3:**
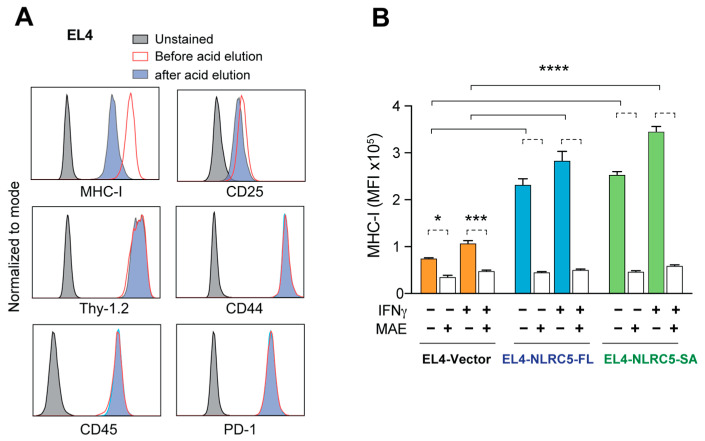
Loss of MHC-I expression in EL4-NLRC5-SA cells following elution of MHC-I-associated peptides (MAPs). (**A**) Selective destabilization of MHC-I expression by acid elution of MAPs. Parental EL4 cells were incubated with citrate phosphate buffer for 2 min, and the expression of MHC-I and the indicated cell surface markers was examined by flow cytometry. Representative data from three experiments are shown. Untreated (red) and unstained samples (grey) cells were used as controls. (**B**) Loss of MHC-I upon acid elution in NLRC5-expressing EL4 cells. EL4 cells expressing empty vector, NLRC5-FL or NLRC5-SA, stimulated or not with 20 ng/mL mIFNγ for 24 h, were subjected to acid elution, and MHC-I expression was measured by flow cytometry. MFI, Mean fluorescence intensity. Mean and standard deviation from three experiments. Turkey’s multiple comparison test: * *p* ≤ 0.05, *** *p* ≤ 0.001, **** *p* ≤ 0.0001.

We have shown that NLRC5-FL expression in B16 melanoma increases the expression of APM genes and the immunogenic potential of tumor cells [36]. This increased tumor immunogenicity could arise from a quantitative increase in tumor antigenic peptides as well as qualitative changes. To characterize these changes, we used mild acid elution (MAE) [48] that destabilizes MHC-I, releasing the associated peptide into the supernatant. To optimize the conditions of elution for MHC-I-associated peptide (MAP) analysis, we used EL4 cells, as their high basal MHC-I expression (Figure 1C) facilitates monitoring the loss of MHC-I. Stripping MAPs from EL4 cells by MAE diminished MHC-I expression, whereas other cell surface proteins such as Thy1.2, CD45, CD25, CD44 and PD-1 were not appreciably affected (Figure 3A). Next, we evaluated the effect of MAE on MHC-I expression in EL4 cells lines expressing NLRC5-FL, NLRC5-SA or vector before and after IFNγ stimulation. IFNγ stimulation caused a discernible increase in MHC-I expression in EL4-NLRC5-FL and EL4-NLRC5-SA cells (Figure 2B). Upon MAE, the MHC-I expression was reduced to a basal level in control as well as IFNγ-treated EL4-vector, EL4-NLRC5-FL and EL4-NLRC5-SA cells to the same extent (Figure 3B), indicating that MAE can be used to assess quantitative and qualitative changes in the MAPs of these cells.

### 2.4. MAP Repertoire of NLRC5-Expressing EL4 Cells

**Figure 4:**
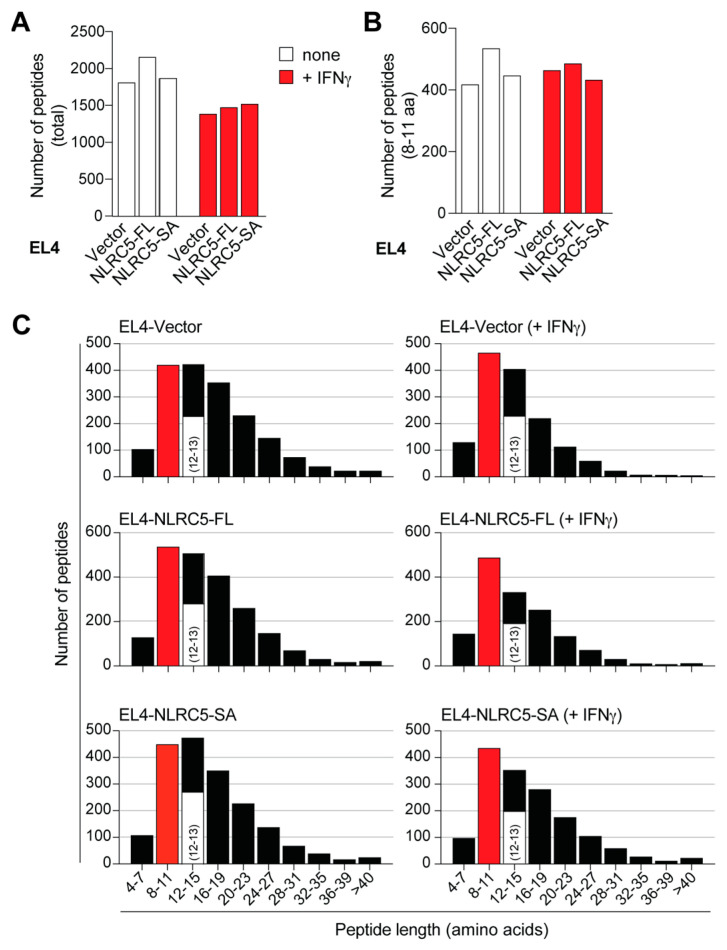
MHC-I-associated peptide repertoire of NLRC5-expressing EL4 cells. (**A**) MAPs were eluted from EL4 cells expressing empty vector, NLRC5-FL or NLRC5-SA, stimulated or not with 20 ng/mL IFNγ for 24 h. The eluted MAPs were identified by the PEAKS software to include peptides from known protein database as well as de novo peptides. Number of all MAPs and 8–11 amino acid long MAPs are shown in (**A**,**B**), and the peptide size distribution in (**C**). The number of 12–13aa long peptides are indicated within the 12–15aa long MAPs. The lists of 8–13aa long peptides from all samples are given in Supplementary Table S2.

MAPs released from EL4-NLRC5-FL, EL4-NLRC5-SA and EL4-vector cells, stimulated or not with IFNγ, were analyzed by mass spectrometry, and the peptide sequences were predicted using the PEAKS software (v8.5) to include de novo peptides that are not represented in known protein databases [49]. Raw mass data of peptide sequences eluted from each cell type are provided in Supplementary Table S1. As shown in Figure 4A, discernibly more peptides were eluted from EL4-NLRC5-FL cells than from EL4-vector cells (2156 versus 1822), whereas EL4-NLRC5-SA cells showed a minimal increase. Compared to unstimulated cells, substantially fewer peptides were eluted from all three cell lines exposed to IFNγ. For example, EL4-NLRC5-FL cells stimulated with IFNγ released 1473 peptides compared to 2156 peptides in non-stimulated cells. A similar trend was observed for EL4-NLRC5-SA and EL4-vector cells. The MHC-I peptide-binding groove predominantly accommodates 8–11aa long peptides [50]. The number of 8–11aa peptides showed a similar trend as the total number of peptides in all three cell lines (Figure 4B); however, the number of peptides eluted from IFNγ-stimulated cells was comparable to that from unstimulated cells.

MAPs are generally isolated by MAE from the cell surface or following immune-affinity purification of MHC-I (MHC-IAP) from cell lysates [51]. A frequent observation when using MAE is the detection of >11aa long peptides [44,51]. Therefore, we plotted all of the identified peptides according to their size. Even though peptides of <8aa (4–7) and >24aa were detected, they were less common than 8–11aa and 12–15aa long peptides (Figure 4C). As some studies have considered 8–13aa peptides for MAP analysis [44], we identified 12–13aa long peptides and grouped them along with 8–11aa peptides for further analysis (Figure 4C). Comparison of 8–13aa peptides showed that 842 MAPs were eluted from EL4-NLRC5-FL cells compared to 691 and 624 from EL4-NLRC5-SA and EL4-vector cells, respectively, under steady-state conditions (Table 1). Notably, 35–45% of the 8–13aa peptides were not derived from known proteins (Table 1), indicating that nearly half of the MAPs at steady state and after IFNγ stimulation arose from non-canonical, unconventional peptides.

### 2.5. NLRC5-SA and NLRC5-FL Differentially Modulate MAPs

**Figure 5:**
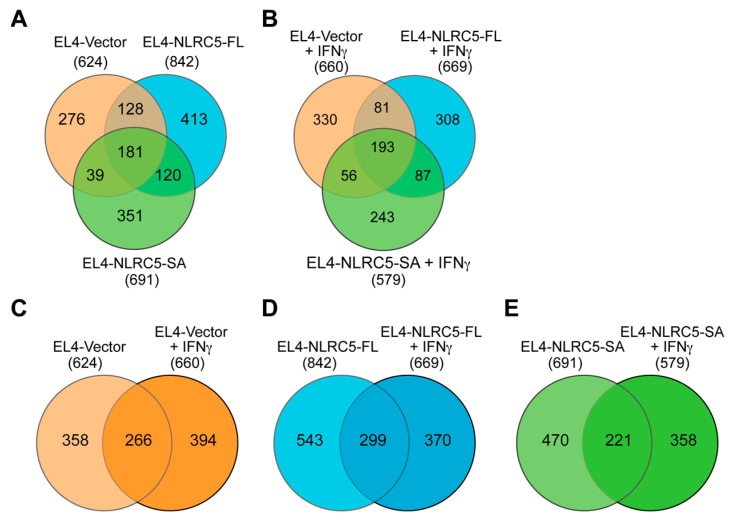
NLRC5-SA and NLRC5-FL differentially modulate MAPs in EL4 cells. (**A**,**B**) Shared and unique 8–13aa MAPs in EL4 cells expressing NLCR5-FL, NLRC5-SA or control vector at steady state (**A**) and after IFNγ stimulation (**B**). The lists of 8–13aa peptides used in (**A**,**B**) are given in Supplementary Table S3. (**C**–**E**) Pairwise comparison of 8–13aa MAPs eluted from unstimulated and IFNγ-stimulated EL4-vector (**C**), EL4-NLRC5-FL (**D**) and EL4-NLRC5-SA (**E**) cells.

Sequence comparison of MAPs eluted from EL4-vector, EL4-NLRC5-FL and EL4-NLRC5-SA cells showed that 181 peptides were shared among all three cell lines, and thus are not modulated by NLRC5-FL or NLRC5-SA (Table 1, Figure 5A). In addition, EL4-NLRC5-FL and EL4-NLRC5-SA cells shared 120 peptides that were not found in EL4-vector cells, indicating substantial overlap in the MHC-I processing pathway modulated by NLRC5-FL and NLRC5-SA. However, several unique MAPs were detected in peptides eluted from EL4-NLRC5-FL (413 peptides) and EL4-NLRC5-SA (351 peptides) cells. A similar trend was observed in the distribution of shared and unique 8–13aa MAPs from IFNγ-stimulated cells, suggesting intrinsic differences between NLRC5-SA and NLCR5-FL in modulating MHC-I antigen processing (Figure 5B). Collectively, these data indicate that even though NLRC5-SA upregulates MHC-I similarly to NLRC5-FL (Figure 1C and Figure 3B), they substantially differ in their ability to modulate MAPs.

IFNγ is a potent stimulator of the MHC-I antigen-processing pathway, which requires the induction of endogenous NLRC5 [30,52,53]. Therefore, we analyzed the 8–13aa peptides eluted from the three cell lines before and after IFNγ stimulation (Figure 5C–E). IFNγ caused substantial changes in the MAP profile of EL4-vector cells, with ~60% (394/660) unique MAPs (Figure 5C). Interestingly, a similar trend was observed in cells expressing NLRC5-SA or NLCR5-FL (Figure 5D,E), suggesting that NLRC5 proteins do not merely recapitulate IFNγ stimulation on the MHC-I antigen presentation pathway, in which case the degree of overlap between unstimulated and IFNγ-stimulated cells would be higher in EL4-NLRC5-FL and EL4-NLRC5-SA cells.

### 2.6. NLRC5-SA Substantially Differs from NLRC5-FL in Modulating MAPs

**Figure 6:**
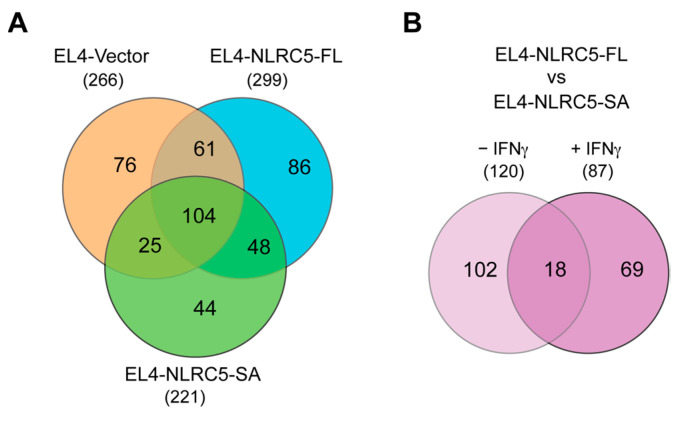
NLRC5-SA significantly differs from NLRC5-FL in modulating steady-state and IFNγ-stimulated MAPs. (**A**) Comparison of 8–13aa MAPs that are not affected by IFNγ stimulation in each of the EL4-derived cell lines. The lists of 8–13aa peptides used in (**A**) are given in Supplementary Table S4. (**B**) The 8–13aa MAPs shared between EL4-NLRC5-FL and EL4-NLRC5-SA cells under steady state or IFNγ-stimulated conditions but absent from EL4-vector cells. The lists of peptides are given in Supplementary Table S5.

To identify MAPs that were presented by all three EL4-derived cell lines, we compared the 266, 299 and 221 peptides, found to be common for steady-state and IFNγ-stimulated EL4-vector, EL4-NLRC5-FL and EL4-NLRC5-SA cells, respectively (Figure 5C–E). This analysis revealed that 104 MAPs were shared among all six samples (Figure 6A), suggesting that they represent MAPs that are constitutively presented and are not influenced by the antigen-processing pathways modulated by IFNγ or NLRC5. Expectedly, there were MAPs exclusively presented by each cell type that were not affected by IFNγ stimulation: 76/266 in EL4-vector, 86/299 in EL4-NLRC5-FL and 44/221 in EL4-NLRC5-SA (Figure 6A). These data indicate that NLRC5 modulates steady-state MAPs that are not affected by IFNγ stimulation; however, NLRC5-SA substantially differs from NLRC5-FL in modulating such steady-state MAPs.

To characterize MAPs that are commonly induced by both NLRC5 constructs, we compared the MAPs shared between EL4-NLRC5-FL and EL4-NLRC5-SA cells under steady state (120) or interferon-stimulated condition (87) that were not shared with EL4-vector cells (Figure 5A,B). Only 18 MAPs were shared between unstimulated and IFNγ-stimulated EL4-NLRC5-FL and EL4-NLRC5-SA cells (Figure 6B), suggesting that these represent MAPs that are not modulated by endogenous NLRC5 induced by IFNγ, but become targets upon overexpression of either NLRC5-FL or NLRC5-SA. Half of these peptides represent unknown sequences, possibly derived from non-coding and non-canonical coding sequences (Table 2). The other MAPs are involved in protein synthesis, RNA processing, chromatin stability, immune responses, signaling pathways and mitochondrial electron transport complex. These data indicate that there is a small but discernible overlap between MAPs induced by NLRC5-FL and NLRC5-SA that are derived from both canonical and non-canonical MAPs.

### 2.7. Potentially Immunogenic NLRC5-SA-Induced MAPs

**Figure 7:**
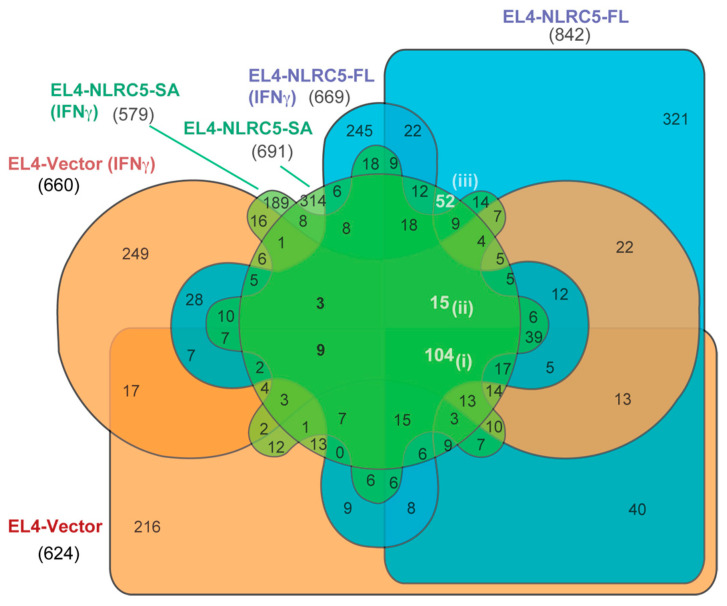
Potential immunogenic MAPs that could be identified using NLRC5-SA. Six-way Venn diagram showing the comparison of MAPs from all the three cell lines at steady state and after IFNγ stimulation.

To assess the commonality and differences among all three cell lines at steady state and after IFNγ stimulation, we made a collective comparison represented by a six-way Venn diagram (Figure 7). This comparison recapitulated the 104 common peptides unchanged by NLRC5 or IFNγ (Figure 7, i; also see Figure 6A). Moreover, 15 peptide sequences (Figure 7, ii) were expressed in all the groups except EL4-vector cells, suggesting that their expression is modulated by IFNγ, presumably via endogenous NLRC5, as they were also present in both EL4-NLRC5-FL and EL4-NLRC5-SA cells before and after IFNγ stimulation. These 15 peptides are listed in Table 3. There were also 52 peptides (Figure 7, iii) presented by the cells expressing either NLRC5-FL or NLRC5-SA but not modulated by IFNγ. The last two groups of MAPs likely include inducers and targets of CD8^+^ T-cell mediated immunity in tumor cells stably expressing NLRC5 that contribute to tumor growth control.

## 3. Discussion

Loss of MHC-I expression is a frequent and key immune evasion mechanism exploited by many cancers to escape CTL-mediated tumor cell killing [54]. As low MHC-I expression and tumor antigen presentation also hampers the outcome of ICB therapies, restoring MHC-I expression in cancers is crucial to reinstate immune-mediated tumor control [28,54,55,56]. Defects in MHC-I expression can result from deletions of one or more HLA alleles (hard lesions) or key antigen-processing pathway genes, or from inhibition of their gene expression by epigenetic mechanisms such as promoter methylation (soft lesions) [15,27,56]. Whereas reversing the hard lesions of MHC-I defects in cancers would require reintroduction of the lost or dysfunctional genes and thus would be difficult to achieve, the soft defects can be reversed by several approaches such IFN stimulation, inhibition of cancer cell signaling pathways that reduce MHC-I expression and epigenetic modifiers. However, these approaches have their own limitations, such as off-target effects, including the induction of the checkpoint blockade molecule PD-L1 by IFNγ [57]. The discovery of NLRC5 as the key transcriptional activator of MHC-I and APM genes that undergoes similar genetic and epigenetic repression in cancers has raised the possibility of using NLRC5 directly to restore MHC-I expression without causing undesirable side effects [58]. However, the large molecular size of NLRC5 restrains its potential immunotherapeutic utility to restore MHC-I expression in cancers. To overcome this limitation, we have generated the NLRC5-CIITA fusion protein NLRC5-SA, which retains the ability to induce MHC-I as efficiently as NLRC5-FL. We show that expressing NLRC5-SA in B16 melanoma and EL4 lymphoma results in efficient tumor control in syngeneic mice. These findings raise the possibility of exploiting NLRC5-SA for cancer gene therapy using viral vectors such adeno-associated or oncolytic viruses to restore MHC-I expression and antitumor immunity in MHC-I-defective tumors that are unresponsive to ICB therapy.

Generation of immunogenic peptides recognized by CTLs is crucial for cancer immune surveillance [59]. In the present study, we compared the impact of NLRC5-FL and NLRC5-SA on MAPs towards exploiting NLRC5 for the discovery of cancer antigenic peptides. Our findings show that both NLRC5 constructs upregulate MHC-I in murine and human cancer cells, promote tumor growth control and induce profound modifications of MAPs that display a partial overlap. The increased efficiency of NLRC5-SA to upregulate MHC-I expression and to cause significant changes in MAPs strengthens the translational potential of NLRC5-SA for cancer immunotherapy.

We used the EL4 cell line, which is extensively used to study MHC-I Ag processing and presentation [44,45,46,47], for the ease of growing in suspension. Despite high basal MHC-I expression, EL4 cells expressing NLRC5-FL or NLRC5-SA showed a discernible increase both at steady state and after IFNγ stimulation. This increase was greater with NLRC5-SA in the human cancer cell lines A549 and T47D, similar to earlier findings on HEK293T cells [43]. It is possible that this increased potency of NLRC5-SA could be intrinsic to its smaller size and increased amount of protein product, or result from its increased transactivation potential, or both. Nonetheless, these findings strengthen the translational potential of NLRC5-SA for restoring tumor immunogenicity and tumor antigen discovery.

Mass-spectrometry-based tumor antigenic peptide discovery requires recovering MAPs following MHC-IAP or by MAE [8,51,60,61,62]. MAE is suitable for suspension cells wherein the peptide elution can be monitored by the loss of MHC-I, whereas MHC-IAP yields more peptides and can be applicable to tumor tissues [48,51,60]. Nonetheless, neither method can be used on cancer cells or tissues that downmodulate MHC-I. NLRC5-SA can be exploited not only to reverse such MHC-I defects but also to maximize the recovery of MAPs as it transactivates genes coding for MHC-I and APM genes. Development of methods for efficient NLRC5-SA delivery to solid tumors, such as adeno-associated viral vectors [43], will be applicable to MAP discovery from solid tumors by MHC-IAP.

Analysis of the MS/MS spectra of peptides eluted from the EL4 cell derivatives yielded thousands of peptides of varying length. Even though the peptide-binding groove of MHC-I predominantly accommodates 8–11aa long peptides, longer peptides of up to 40aa can be transported across the endoplasmic reticulum and up to 18aa long peptides could be accommodated by MHC-I [63,64]. Even though 8–13aa long peptides constituted the bulk of the MAPs from all three cell lines, we also observed longer peptides of varying lengths. These longer peptides appear to be a frequent occurrence with MAP isolation by MAE, as they were also observed by other groups and are generally considered as contaminants [44,51]. Therefore, MAPs in the range of 8–13aa were sorted and analyzed further for comparing NLRC5-FL and NLRC5-SA in modulating the MAP repertoire. MAPs are in general fewer in number than the overall proteome of a cell, as the majority originate from rapidly degraded proteins with a shorter half-life [65,66]. MAPs are generated by proteasomal cleavage of not only the known proteins from coding sequences but also from the defective ribosomal products (DRiPs) arising from out-of-frame start codons, alternatively spliced RNA and 3′-untranslated regions that are outside the coding region [7,61,67,68,69,70,71,72,73]. Moreover, proteasomes catalyze peptide splicing, resulting in new MAPs [74,75]. These non-canonical ‘cryptic’ MAPs are believed to contribute as much to antitumor immune surveillance as the conventional MAPs [10]. Efforts are being made to detect such non-canonical MAPs [7,9,76,77], as the cryptic MAPs will not be identified by Mascot and Maxquant tools, which rely on UniProtKB and ENSEMBL databases of known proteins [78,79]. Therefore, we used the PEAKS software, which predicts peptide sequences from raw mass spectra, allowing the identification of previously unknown sequences [49]. Our results show that 35–45% of 8–13aa MAPs from the three EL4-derived lines represent cryptic MAPs, highlighting the need to identify their source and importance in antitumor immunity. Development of databases such as the in silico translated transcriptome in three different reading frames and in both directions will advance progress in this area [7,9].

NLRC5-SA induced MHC-I expression to a level comparable to NLRC5-FL in EL4 cells and in human cancer cells. The 8–13aa MAP repertoire induced by NLRC5-SA was only marginally (~15%) smaller than that induced by NLRC5-FL and showed substantial overlap at steady state and after IFNγ stimulation (Figure 4B,C). However, the MAP repertoire uniquely induced by NLRC5-SA and NLRC5-FL suggests that NLRC5-FL and NLRC5-SA may differ in their ability to modulate the MHC-I APM due to different C-terminal LRRs. In this context, it is noteworthy that NLRC5-SA also retains the ability to upregulate MHC-II genes [43], which could promote activation of antitumor CD4^+^ T cells and potentiate the overall antitumor immune response.

Overall, our findings indicate that NLRC5-SA can be exploited in tumor immunotherapy approaches for diverse cancers in two ways. Firstly, NLRC5-SA could be introduced into tumors using viral vectors to restore MHC-I expression and upregulate antigen processing and presentation to elicit protective antitumor immunity [58]. Secondly, NLRC5-SA could be exploited for the discovery of tumor antigenic peptides from primary tumor cells and tumor organoid cultures. Dominant tumor antigenic peptides could then be used for loading autologous dendritic cells and mesenchymal cells and artificial APCs to elicit antitumor immune responses [80,81,82,83].

## 4. Materials and Methods

### 4.1. Cell Culture

B16.F10 melanoma cells expressing vector or NLRC5-FL were previously reported [36]. EL4 cells (TIB-39^™^) obtained from the American Type Culture Collection (Manassas, VA, USA) were grown in RPMI medium (Multicell, Tucker, GA, USA) containing 10% fetal calf serum (FCS, Gibco, CA, USA), 100 U/mL penicillin/streptomycin, 1 mM sodium pyruvate and 10 mM HEPES (Multicell). Human cancer cells A549 (lung, ATCC cat# CCL-185^™^), MCF7 (breast, ATCC cat# HTB-22^™^) and T47D (breast, ATCC cat# HTB-133^™^) were kindly provided by Dr. Claire Dubois (Université de Sherbrooke, Canada). These A549, MCF7 and T47D cells were grown in RPMI-10% FCS and other supplements as for EL4 cells, and 0.1 Units/mL of insulin.

### 4.2. Plasmids and Derivation of NLRC5-Expressing Lines

Human eGFP, eGFP-NLRC5-FL and eGFP-NLRC5-SA expression constructs in pCMV-Tag2B vector are described by Neerincx et al. [43,84]. EL4 cells were transfected with NLRC5-FL, NLRC5-SA and empty vector by electroporation using the Amaxa Cell Line Nucleofector Kit (Lonza, Switzerland). Human cancer lines were transfected using Lipofectamine 3000 (Thermo-Fisher, Waltham, MA, USA). Stable cell lines were selected using 1–2 mg/mL G418 (Wisent, QC, Canada), cloned by limiting dilution, and GFP-positive wells were pooled to establish EL4 cell lines expressing NLRC5-FL, NLRC5-SA or the control vector. A similar strategy was used for establishing human cancer cells expressing the different vectors and B16.F10 cells expressing NLRC5-SA.

### 4.3. IFNγ Stimulation and Flow Cytometry

EL4 cells expressing NLRC5-FL, NLRC5-SA or control vector were treated with mouse IFNγ (20 ng/mL, Peprotech, Rocky Hill, NJ, USA) for 24 h or left untreated. To detect the expression of surface markers, cells suspended in PBS containing 2% FCS were stained with fluorochrome-conjugated antibodies obtained from Biolegend (San Diego, CA, USA) or eBiosciences (San Diego, CA, USA) listed in Table 4. The stained cells were analyzed using CytoFLEX (Beckman Coulter, CA, USA) as previously described [36]. Statistical analysis was carried out using the GraphPad Prism 8 Software (San Diego, CA, USA).

### 4.4. Tumor Growth

Eight-week-old C57BL/6 mice (Jackson Laboratory, Bar Harbor, ME, USA) were injected subcutaneously with 1 × 10^5^ B16-vector, B16-NLRC5-FL or B16-NLRC5-SA cells, or 5 × 10^5^ EL4-vector, EL4-NLRC5-FL or EL4-NLRC5-SA cells. Tumor growth was monitored every 2–3 days. Tumor volume was calculated using the formula: 1/2 (length × width^2^). Animal experiments were approved by the Université de Sherbrooke Ethics Committee for Animal Care and Use (Protocol #2019-2206).

### 4.5. Mild Acid Elution and Purification of MHC-I-Associated Peptides

MHC-I-associated peptides (MAPs) were collected from untreated and IFNγ-treated EL4-NLRC5-FL, EL4-NLRC5-SA and EL4-vector cells by mild acid elution (MAE) [48]. From three independent cultures for each cell type, 30 × 10^6^ cells were washed in PBS before adding citrate phosphate (CP) buffer (0.131 M citric acid, 0.066 M Na_2_HPO_4_, 0.15 M NaCl, pH 3.3) containing 10 mM iodoacetamide and 1 µg/mL aprotinin for 2 min. The cells were centrifuged and the supernatant containing peptides was collected. Aliquots of cells collected before and after MAP elution were evaluated for the loss of MHC-I.

MAPs in supernatants were concentrated using Oasis HLB cartridges (30 mg, 1 cc, Waters, MA, USA) following published methods [44]. Briefly, the HLB cartridges were conditioned with 100% methanol followed by water wash under positive pressure before passing the samples to bind MAPs. The bound MAPs were eluted using 2 mL of 80% methanol in water containing 0.2% formic acid (FA). The eluent was diluted to 40% methanol in water containing 0.2% FA. Size exclusion of peptides was done using 10 kDa Amicon ultrafiltration devices (Millipore, MA, USA) to remove β2M (13 kDa), which is destabilized along with the MHC-I alpha chain. The resulting flow-through was dried using a SpeedVac vacuum concentrator, resuspended in 25 μL of 1% FA and peptide concentration was determined using the Nanodrop (Thermo-Fisher) at 205 nm.

### 4.6. Elution of MAPs and Mass Spectrometry

Eluted peptides were purified and concentrated, and 2 µg of the peptides was subjected to mass spectrometry analysis using an OrbiTrap QExactive mass spectrometer (Thermo Fisher Scientific) at the Proteomics platform of the Université de Sherbrooke as detailed in supplementary methods [85]. Briefly, 2 µg total peptides were separated using a nanoHPLC system (Dionex Ultimate 3000) and loaded with consistent flow of 4 µL/min onto an Acclaim PepMap100 C18 Trap Column (0.3 mm i.d. × 5 mm, Dionex Corporation, Sunnyvale, CA, USA). The peptides were eluted onto a PepMap C18 analytical nano column (75 µm × 50 cm, Dionex Corporation) with a linear gradient of 5–35% solvent B (0.1% FA and 90% acetonitrile) for more than four hours with flowrate of 200 nL/min. EasySpray source, with 2.0 kV spray voltage, was coupled to Orbitrap Mass Spectrometer with the column temperature set at 40 °C. A full scan of survey spectra of *m*/*z* 350–1600 was acquired at a resolution of 70,000 after amassing 1 million ions in profile mode. Fragmenting of 10 most intense peptide ions was made by collision-induced dissociation. Unassigned charge states including single, 7 and 8 charged species were excluded by screening the precursor ion charge state. Mass accuracy was improved by enabling the lock mass option for survey scan. Xcalibur™ software (Thermo Scientific) was utilized for data acquisition. The resulting mass spectra were analyzed through PEAKS Studio X software (v8.5, Bioinformatics Solutions Inc., Waterloo, ON, Canada) [49].

### 4.7. Data Analysis and Mining

Mass spectrometry data from IFNγ-non-treated and treated EL4-NLRC5-FL, EL4-NLRC5-SA and EL4-vector cells from two biological replicates were analyzed. Data from the one of the replicates was omitted due to very low mass spikes and very few peptides identified. All identified peptides were tabulated and sorted to determine the MAP repertoire of each EL4-derived cell line. Raw mass spectrometry data of peptide sequences eluted from each cell type are provided in Supplementary Table S1. Only 8–13 amino acid (aa) long peptides were considered to study the shared and unique MAP repertoire using the InteractiVenn webtool [86].

Expression of NLRC5, MHC-I and APM genes in the National Cancer Institute cell line panel NCI-60 was analyzed using the CellMiner™ database [87,88]. Z-scores were used to compare expression of query genes and to calculate correlations between gene expression.

### 4.8. Statistical Analysis

Data were analyzed using the GraphPad Prism9 (San Diego, CA, USA). Statistical significance (*p* value) was calculated by two-way ANOVA with Tukey’s multiple comparison test, and *p* values < 0.05 were considered significant.

## 5. Conclusions

NLRC5-SA upregulates MHC-I in mouse and human cancer cells as efficiently as full-length NLRC5. NLRC5-SA promotes tumor growth control similarly to full-length NLRC5. NLRC5-SA induces significant modulation of the MHC-I peptide repertoire in tumor cells that partially overlaps with changes induced by full-length NLRC5. Thus, NLRC5-SA can be used to overcome the size constraint of full-length NLRC5 for delivery to MHC-I defective tumors via viral vectors to induce tumor immunogenicity and to identify tumor antigenic peptides for immunotherapy applications.

## Figures and Tables

**Table 1:** Number of known and unknown 8–13aa long MAPs identified in each cell type.

Cell Population	Total Peptides	Known	Unknown	% Unknown
EL4-Vector	624	374	250	40%
EL4-NLRC5-FL	842	543	299	35%
EL4-NLRC5-SA	691	381	310	45%
EL4-Vector (IFNγ)	660	367	293	44%
EL4-NLRC5-FL (IFNγ)	669	357	312	46%
EL4-NLRC5-SA (IFNγ)	579	324	255	44%

**Table 2:** Identity of the 18 peptides shared between NLRC5-FL- and NLRC5-SA-expressing EL4 cells in IFNγ-independent fashion. Numbers in brackets indicate the increase in aa mass due to post translational modification.

Peptide	Length	Accession Name	Protein Functionality in	−10lgP	*m*/*z*
KVFLENVIRDA	11	Unknown	-	57.42	435.2499
KVFLENVIRD	10	Unknown	-	57.26	411.5716
VVLRNPLIAGK	11	SMD2	Pre-mRNA splicing	56.32	393.9244
S(+42.01)GGLLKALRSDSY	13	NCBP2	RNA processing	54.98	704.8751
AKALANVNIGSL	12	RLA1	Protein synthesis	49.39	585.8455
AVRLLLPGEL	10	Unknown	-	45.61	540.8425
AVRLILPGEL	10	Unknown	-	45.61	540.8425
RFQSSAVM(+15.99)AL	10	H3.1	Chromatin stability	45.21	563.2897
SVEDIHSRFQSL	12	ILEUA	Innate immune response	42.6	473.2405
LIRKLPFQRL	10	H3C	Chromatin stability	37.55	428.616
LLRKLPFQRL	10	Unknown	-	37.55	428.616
KQQIATFDT	9	Unknown	-	31.22	526.2757
FAKALANVN	9	RLA1	Protein synthesis	30.9	474.2691
DTLSEESYKDSTL	13	1433Z	Adapter protein in regulation of multiple signaling pathways	28.1	744.3399
LVLPVPAF	8	Unknown	-	28.06	428.2725
IVLPVPAF	8	Unknown	-	28.06	428.2725
LAGTEAVVEAI	11	Unknown	-	27.65	536.7983
LEVQGRDSRLVL	12	ATPB	Mitochondrial electron transport complex	25.71	462.2681

**Table 3:** Fifteen peptides commonly being presented by all EL4 cell types excluding EL4-vector cells. Numbers in brackets indicate the increase in amino acid mass due to possible post translational modification.

Peptide	Length	Accession Name	Protein Functionality in	−10lgP	*m*/*z*
VREIAQDFKTDL	12	H3C	Chromatin stability	56.04	478.9242
DRLHISPDRVY	11	MIF	Innate immune response	54.61	457.5773
A(+42.01)EDIKTKIKNYK	12	CX6B1	Mitochondrial electron transport complex	53.05	498.2838
SIRGNNIRY	9	SMD1	Pre-mRNA splicing	52.53	364.8692
SLRGNNIRY	9	Unknown	-	52.53	364.8692
VYIKHPVSLEQYL	13	PSMD8	Proteasomal processing	47.32	530.296
PRKIEEIKDFL	11	RL38	Protein synthesis	46.74	463.2697
AAVLEYLTAE	10	H2A1	Chromatin stability	45.46	540.2855
FASPTQVF	8	ATPD	Mitochondrial electron transport complex	44.68	896.4516
IEDDKSRLVL	10	MA2B2	Spermatogenesis	44.44	396.5593
SLVYPFPGPIPN	12	Unknown	-	40.72	650.8497
LAEEAVTLD	9	ATPD	Mitochondrial electron transport complex	39.51	960.4878
TGPSNVDKL	9	CHK1	Cell cycle regulation	37.12	465.7493
PLRAQQLAAEL	11	TIM10	Mitochondrial intermembrane chaperone	35.53	605.351
T(+42.01)RDFKPGDLIFA	12	PSIP1	Stress-induced apoptosis	27.11	711.3745

**Table 4:** Antibodies used for flow cytometry.

Marker	Antibody Clone	Conjugate	Source	Catalog Number
H-2K^b^/D^b^	28-8-6	Alexa 647	Biolegend	114612
CD45	30-F11	BV605	Biolegend	103140
CD44	IM7	PE Cy7	eBiosciences	25-0441-82
CD25	3C7	FITC	Biolegend	101908
Thy 1.2	30-H12	APC	Biolegend	105312
PD-1	J43	PE	BD	551892
HLA-ABC	W6/32	PE	eBiosciences	12-9983-42

## Data Availability

All data generated or analyzed during this study are included in the supplementary information files.

## Supplementary Materials

The following supporting information can be downloaded at: https://www.mdpi.com/article/10.3390/ijms24087206/s1.
