# Supplementary material for: NLRC5-CIITA Fusion Protein as an Effective Inducer of MHC-I Expression and Antitumor Immunity
Source: Int J Mol Sci. 2023 Apr 13;24(8):7206. doi: 10.3390/ijms24087206 (PMC10138588; doi:10.3390/ijms24087206)
Supplement: Supplementary file 1 [file ijms-24-07206-s001.zip › Supplementary figures.pdf]

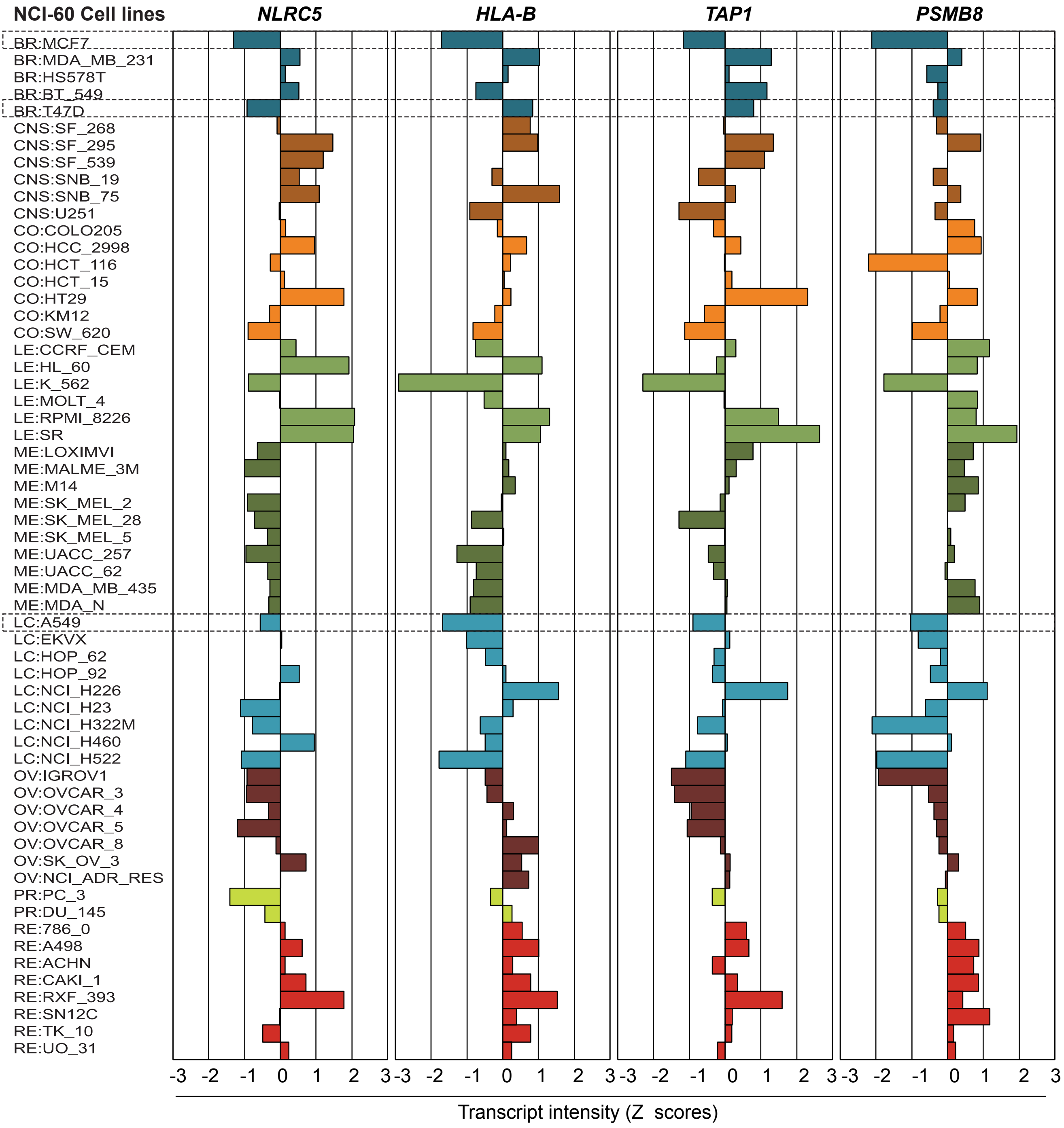

Supplementary Figure S1. Expression of NLRC5 and MHC-I pathway genes in the NCI-60 panel of cancer cell lines. The transcriptomic data on the NCI-60 panel of cancer cell lines from the CellMinerTM database was queried to evaluate the expression of NLRC5, HLA-B, B2M, PSMB8, PSBM9, PSMB10, PSME1, PSME2, TAP1, TAPBP and STAT1 genes. Graphic representation of the z-scores for the expression levels of NLRC5, HLA-B, PSMB8 and TAP1 are shown. BR: Breast cancer; CNS: Central Nervous System; CO: Colon cancer; LE: Leukemia; ME: Melanoma; LC: Lung cancer; OV: Ovarian Cancer; PR: Prostate cancer; RE: Renal Cancer.

A

		MHC-I		Proteasome components					Peptide transport & loading		
			β2m	LMP7	LMP2	MECL1	PA28α	PA28β		Tapasin	
	NLRC5	***HLA-B***	***B2M***	***PSMB8***	***PSMB9***	***PSMB10***	***PSME1***	***PSME2***	***TAP1***	***TAPBP***	***STAT1***
NLRC5	**1**										
HLA-B	**0.56**	**1**									
B2M	**0.471**	0.271	**1**								
PSMB8	**0.572**	**0.55**	**0.445**	**1**							
PSMB9	**0.746**	**0.61**	**0.58**	**0.837**	**1**						
PSMB10	**0.587**	0.265	**0.406**	**0.595**	**0.686**	**1**					
PSME1	0.28	**0.458**	0.19	**0.452**	**0.504**	**0.478**	**1**				
PSME2	**0.337**	**0.342**	0.304	0.307	**0.516**	**0.368**	**0.727**	**1**			
TAP1	**0.677**	**0.672**	**0.413**	**0.654**	**0.795**	**0.356**	**0.457**	**0.528**	**1**		
TAPBP	**0.368**	**0.583**	0.285	**0.483**	**0.625**	0.298	**0.538**	**0.464**	**0.719**	**1**	
STAT1	**0.418**	**0.41**	**0.374**	0.324	**0.501**	0.189	0.245	0.28	**0.549**	**0.498**	**1**

B

**HLA Expression**	**NLRC5 Expression**	**Cell lines**
Very Low (---)	Very Low (---)	BR: MCF-7
Low (-)	Very Low (---)	PR: PC3
Very Low (---)	Low (-)	LE: K-562, LC: A549, LC: NCI-H522,
High (+)	Low (-)	BR : T47D, LC: NCI-H226

Supplementary Figure S2. (A) Correlation between the expression of NLRC5 and MHC-I and the antigen processing machinery genes in the NCI-60 panel cell lines. Pearson's correlation between the expression levels of NLRC5 and the MHC-I antigen presentation pathway genes. Values shown in bold represent significant positive correlations when n = 35 and $p < 0.05$ with r = 0.334, in the absence of multiple comparisons correction. (B) Patterns of NLRC5 and HLA mRNA expression levels in NCI-60 cell lines and examples.
